# Technologies monitoring and improving biosecurity compliance in barn anterooms

**DOI:** 10.3389/fvets.2022.1005144

**Published:** 2022-11-04

**Authors:** Manon Racicot, Anne-Marie Cardinal, Dominic Tremblay, Jean-Pierre Vaillancourt

**Affiliations:** ^1^Department of Pathology and Microbiology, Faculty of Veterinary Medicine, Université de Montréal, Montreal, QC, Canada; ^2^Department of Clinical Sciences, Faculty of Veterinary Medicine, Université de Montréal, Montreal, QC, Canada; ^3^Institut de technologie Agroalimentaire du Québec, Programme de technologie des productions animales, St-Hyacinthe, QC, Canada

**Keywords:** biosecurity, compliance, technology, RFID, traffic, monitoring, anteroom

## Abstract

People can act as mechanical vectors, and introduce and spread infectious diseases on farms. Preventive measures, such as changing boots and washing hands, need systematic implementation to manage this risk. Unfortunately, biosecurity compliance regarding biosecurity measures in barn anterooms has been shown to be generally low in all animal production systems. Indeed, the main challenge with biosecurity is maintaining compliance. The development of an effective on-farm biosecurity program requires several elements. These include farm and barn designs facilitating implementation of biosecurity measures; consistently communicating with all personnel and visitors informing them about threats and biosecurity; training programs for all farm personnel, explaining why biosecurity is effective in preventing infectious disease transmission, which measures are needed, and how to best implement them. All these components would be further optimized if automated monitoring systems were implemented with feedback mechanisms. Technologies are now available and are being adapted to the farm context to monitor biosecurity compliance. Two pilot projects using radio-frequency-identification-based (RFID) real-time continuous automated monitoring system quantifying hand sanitizing and boot compliance were conducted. The first one (*MediHand Trace* system) was a system designed to monitor and provide real-time feedback for handwashing in a hospital environment. It was functional for this task, although not sturdy enough for long-term use in a farm environment. The second system was a prototype designed for barns and with foot mats allowing the monitoring of footwear management as well as handwashing. These pilot studies have shown that real-time feedback helps improve compliance. However, the efficacy of the systems was very dependent on the physical set-up of the anteroom.

## Introduction

Biosecurity is a health strategy, a dynamic balance between a host and its environment, aimed at protecting an animal population from transmissible infectious agents (1). It reduces the risk of introducing diseases (2) and reduces financial losses following an infection (3). It is defined by the management, prevention and monitoring of diseases and includes efforts, planning and strategies to protect animals, humans and the environment. It includes infrastructure (e.g., Danish entry), technologies (e.g., test assessing environmental contamination), techniques (e.g., vermin control) and hygiene practices (e.g., changing of boots and washing hands). It also includes communications to manage the movement of animals, personnel and equipment. It is therefore the sum of risk management strategies preventing the introduction, exposure and transmission of hazards in a population. Following the development and implementation of a biosecurity protocol, the major challenge remains the constant and daily application of these measures.

Several studies have identified people-related biosecurity measures as risk factors for multiple diseases (4–13). For example, the lack of a hygiene barrier in broiler barn entrance increases the chances of *Campylobacter* contamination by more than three times (14). Van de Giessen et al. (15) reported that chances of *Campylobacter* contamination are reduced by five times when people wash their hands, change farm boots and use footbath. On-farm biosecurity was also demonstrated to be important for managing *Salmonella* spp. (16). A study on infectious bronchitis in Quebec, Canada showed that failure to change boots between barns increased the risk of infection 10-fold (J-P Vaillancourt, personal communication). An investigation of infectious laryngotracheitis in broilers in the Niagara Peninsula in Ontario also showcased the importance of proper biosecurity for visitors (17). Similar results are reported for other important conditions such as mycoplasmosis and avian influenza (18–20).

However, these simple preventive measures are unfortunately not consistently followed. A study by Racicot (21–23) described the application of biosecurity measures at the entrance and exit of 24 poultry barns in Quebec, Canada, using hidden cameras. Results showed that when the barn entrance design is difficult to comply with, there are 13 times fewer chances to comply with the hygiene barrier. The type of hygiene barrier has also a significant impact on compliance. A bench being preferable to a simple line on the floor. Furthermore, boot and handwashing compliance were only at 53 and 36%, respectively. These failures to prevent the introduction of pathogens at the farm level pose a significant risk for the entire poultry production chain, and ultimately to trade and consumers. The impact of these behavioral failures was evaluated in an experimental study where phage and genetically modified bacteria (detectable by bioluminescence) were used to assess the level of floor and boot contamination. Results show that when performing the right behavior, contamination is prevented (24).

Racicot et al. (21) reported a total of 44 different biosecurity mistakes observed from 883 visits done by 102 different individuals using hidden cameras. On average, four errors were recorded per visit. People observed over several visits made on average six different errors. Twenty-seven out of the 44 errors (61.4%) were related to area delimitation (clean vs. dirty), six to boots (13.6%), five to hand washing (11.4%), three to coveralls (6.8%) and three to logbooks (6.8%). Interestingly, people tend to repeat the same mistakes over time. This is in agreement with another study done on dairy farms where milking procedures were observed. It was found that producers were consistent in the application of milking procedures across time, regardless of whether or not they were correct (25).

Haynes et al. (26) describe different strategies to improve compliance with medical recommendations. The simple fact of tailoring the medical recommendation with a daily habit is an effective way to increase compliance. In addition, frequent reminders to follow recommendations increased compliance from 24 to 70%. However, there is a decline over time. According to Conrad (27), two social domains emerge to explain the issue of compliance: communication and beliefs. Thus, appropriate instructions with clear information and feedback should improve compliance. The majority of the strategies evaluated aim to improve handwashing of healthcare personnel. A study reveals that handwashing compliance does not increase with the experience of healthcare personnel, but it can be improved following the implementation of a training program. After its implementation, compliance with hand washing before and after contact with patients increased from 13 and 15% to 73 and 81%, respectively. However, it declines over time, dropping to 26 and 23% after 4 years (28). Thus, training programs alone are not enough to maintain long-term adherence. Feedback and reinforcement are necessary. Indeed, the same authors demonstrated the effectiveness of feedback on handwashing frequency: compliance increased from 63 to 92% for 3 weeks. Other studies have also demonstrated the effectiveness of frequent feedback in improving handwashing compliance (29, 30). Tibballs (31) reports that using feedback, handwashing increases from 12.4% and 10.6% to 68.3% and 64.8% before and after contact with a patient, respectively. Seven weeks after the feedback stops, handwashing decreases to 54.6 and 54.9%, which is still higher than the initial compliance. Hence, feedbacks are needed to maintain compliance with a desired behavior (32). However, the main challenge with on-farm biosecurity is to evaluate compliance of daily behaviors, such as changing boots and washing hands when entering a barn, for which compliance, using video surveillance, is as low as 53 and 36%, respectively (21).

New technologies are now available in hospitals for monitoring compliance and providing regular feedback to employees and visitors. Two pilot studies were conducted to determine whether a similar approach would work under farm conditions. Hence, the objective of these studies was to adapt and assess, under farm conditions, the impact on compliance of radio-frequency-identification-based (RFID) real-time continuous automated monitoring systems.

## Materials, methods, and results

### Pilot project using the *MediHandTrace* system

A RFID continuous automated monitoring system has been developed to monitor compliance with hygienic behavior and provide regular feedback, in-real time and/or through pre-defined scheduled short message services (SMS) (e.g., weekly). When evaluated against video recordings in a hospital environment, the RFID system was found to be accurate (99.02%), sensitive (95.65%) and specific (100%) (33). This system was successfully tested and implemented again in a hospital context to monitor handwashing compliance of health care professionals (34).

In 2018, this RFID technology was adapted to quantify boot and hand sanitizing compliance when entering and exiting a barn, although it was not designed for poultry farm conditions. The main objective of this pilot project was to confirm the adaptability of the *MediHandTrace* (MHT, La Garde, France) technology in a farm production environment. The study also aimed at monitoring and evaluating the frequency of two biosecurity measures: hand washing and changing of boots according to the different biosecurity areas (clean/dirty) during a visit to a barn, and providing real-time feedback and employee performance evaluations. As the project was exploratory, a convenient sample of two farms near the Faculty of Veterinary Medicine in St-Hyacinthe, Quebec, Canada was chosen. Farms had to have a hygiene barrier in their barn entrances (red line or a bench) and require the targeted biosecurity measures. The selected barn had to have animals in production at the time of the study.

Employees were asked to participate on a voluntary basis. As per the Université de Montréal ethic approval (certificate #2018-688, CERES-18-097-D), a consent form was completed by each participant. Recruitment was done in person. Explanations were given verbally and supported by written documents specifying in detail the purpose of the study as well as how to proceed. Diagrams and poster describing the steps to follow when entering a barn were also presented, and then installed in the barn entrances of participating farms throughout the study.

**Figure 1:**
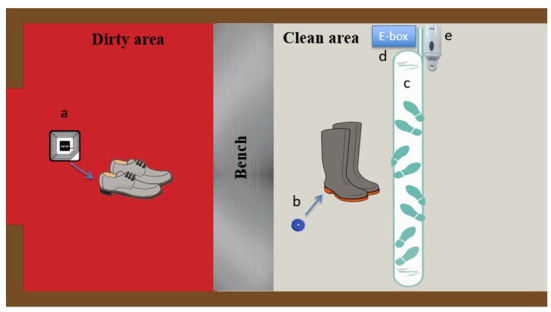
*MediHandTrace* adapted Radio-frequency-identification-based (RFID) real-time continuous automated monitoring system. **(a)** Soft chips inserted in work shoes used by personnel getting to the farm; **(b)** rigid chips inserted in the soles of farm boots; **(c)** RFID antenna; **(d)** *MediHandTrace* device (e-box) with data management program; **(e)** hand sanitizer device.

Each employee was assigned a pair of farm boots with a rigid chip inserted in the sole of each boot for the duration of the project. With their approval, soft chips were inserted under the sole of the shoes that participants used to come to work. Each employee could then be assessed individually during their barn visits. Visitors were asked to wear specifically identified boots. Identification of the visitors was done using a logbook. At the end of the observation period, the chipped boots were recovered by the research team and the chips implanted in the participants' shoes were removed. The data were collected using the RFID system equipped with an antenna installed on the floor of the clean area and connected to the alcohol-based sanitizer, detecting hand sanitization and chipped farm boots (Figure 1). Detection of chipped farm boots by the antenna located in the clean area indicated farm boot compliance, while detection of chipped personal shoes indicated lack of compliance. Because the system only had one antenna in the clean area, it was able to monitor boot compliance when entering a barn, but not when exiting. Indeed, the system could not detect if farm boots were removed in the right area (e.g., farm boots removed in the clean area) or if they were worn outside the barn. To do so, another antenna would have been needed in the dirty area, allowing an alarm to be triggered when farm boots would have been detected by this antenna.

Detection of chipped farm boots or personal shoes by the antenna (entering or leaving the barn) without pressure detected on the hand sanitizer device indicated a lack of compliance with hand hygiene. The system was thus able to monitor hand sanitizing compliance when entering and exiting the barn. In the event of a breach in applying this biosecurity rule, an immediate feedback *via* a sound alarm was sent by the system reminding people to comply. The system was also designed to monitor if the biosecurity breach was corrected after hearing the alarm. The MediHandTrace device (e-box) had a data management program that allowed data collection *via* a USB key. Data were extracted at the end of the study period.

This RFID system was tested on two farms in 2019: an egg layer farm and a broiler farm. A questionnaire was also designed to evaluate employees' appreciation of the system.

For the egg layer farm, 254 entries and exits (i.e., 127 visits) by four different employees were recorded between February 18 and March 6, 2019 (17 days). The average number of entries and exits per day was 15, with no difference between the week days and the weekend. Boots were donned in the clean area 122 times out of 131 opportunities (93%). On four visits, boots were changed twice during a same visit. Hands were sanitized 173 times out of 254 opportunities (68%). Three out of four employees fully complied with boot change as their personal shoes were not detected by the RFID system during the entire trial. For the 9 non-compliant shoe events, farms boots were changed twice (2/9; 22%) after hearing the alarm. Out of the 81 non-compliant hand sanitizing occurrences, 26 (32%) were corrected after the alarm rang. The alarm improved hand sanitizing compliance by 2.5–25% at the individual level. The individual who did not get 100% compliance for changing boots also had a high compliance for handwashing (73%), which increased by 10% once the alarm system was activated. The individual with the lowest handwashing compliance rate (16.8%) had the highest improvement (25%) after hearing the alarm. The employee with the highest handwashing compliance rate (91.7%) was not influenced by the alarm.

For the broiler farm, 56 entries and exists (i.e., 28 visits) by three different employees were recorded between May 27 and June 8, 2019 (13 days). The average number of entries and exits per day was 5 for the week days and 2 for the weekend. Boot compliance was at 100% for the entire period and hand sanitizing compliance was at 73.2% (41 hand sanitizing events out of 56 opportunities). Out of the 15 non-compliant hand sanitizing occurrences, one was corrected after the alarm rang.

**Figure 2:**
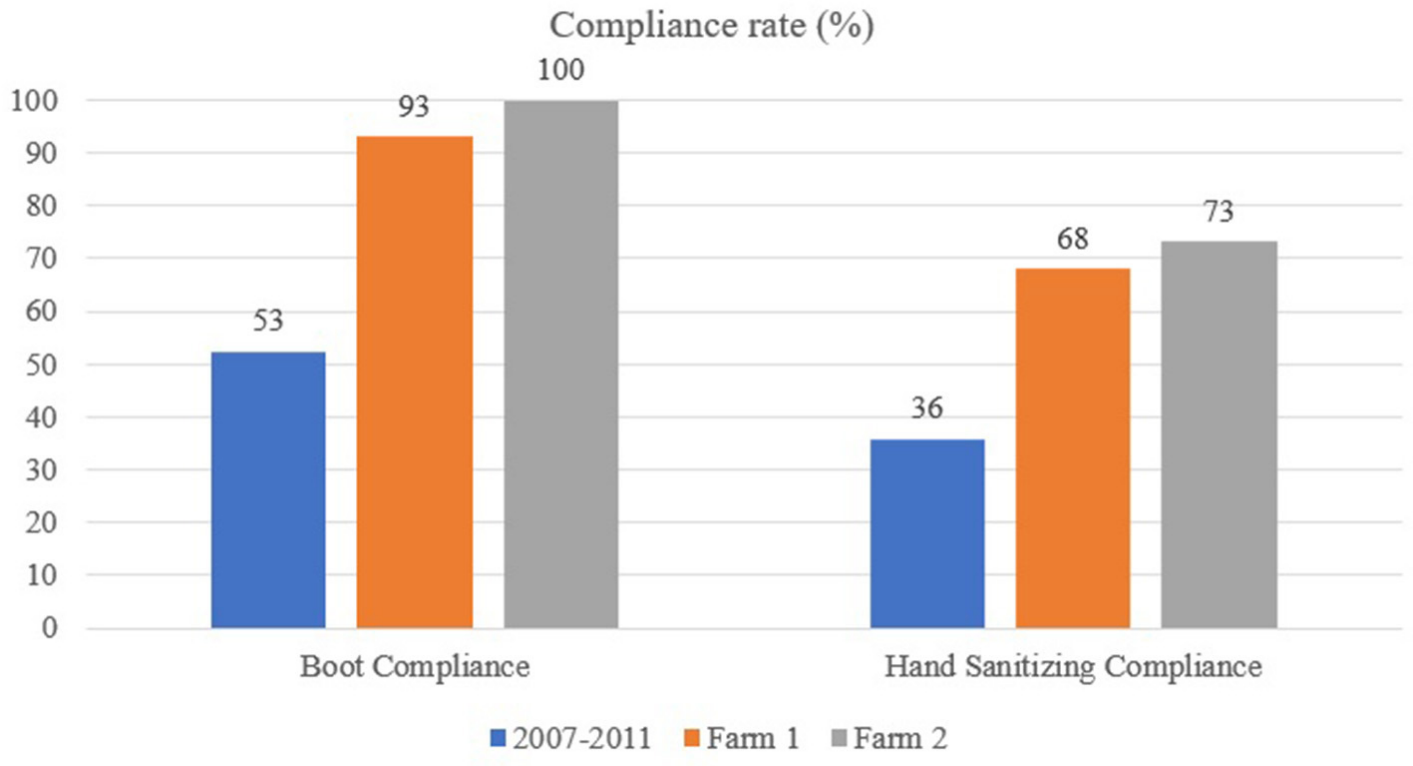
Boot and hand sanitizing compliance at the farm level using the *MediHandTrace* system: comparison between Racicot et al. (21) study (performed between 2007 and 2011), egg layer farm (Farm 1) and broiler farm (Farm 2).

**Figure 3:**
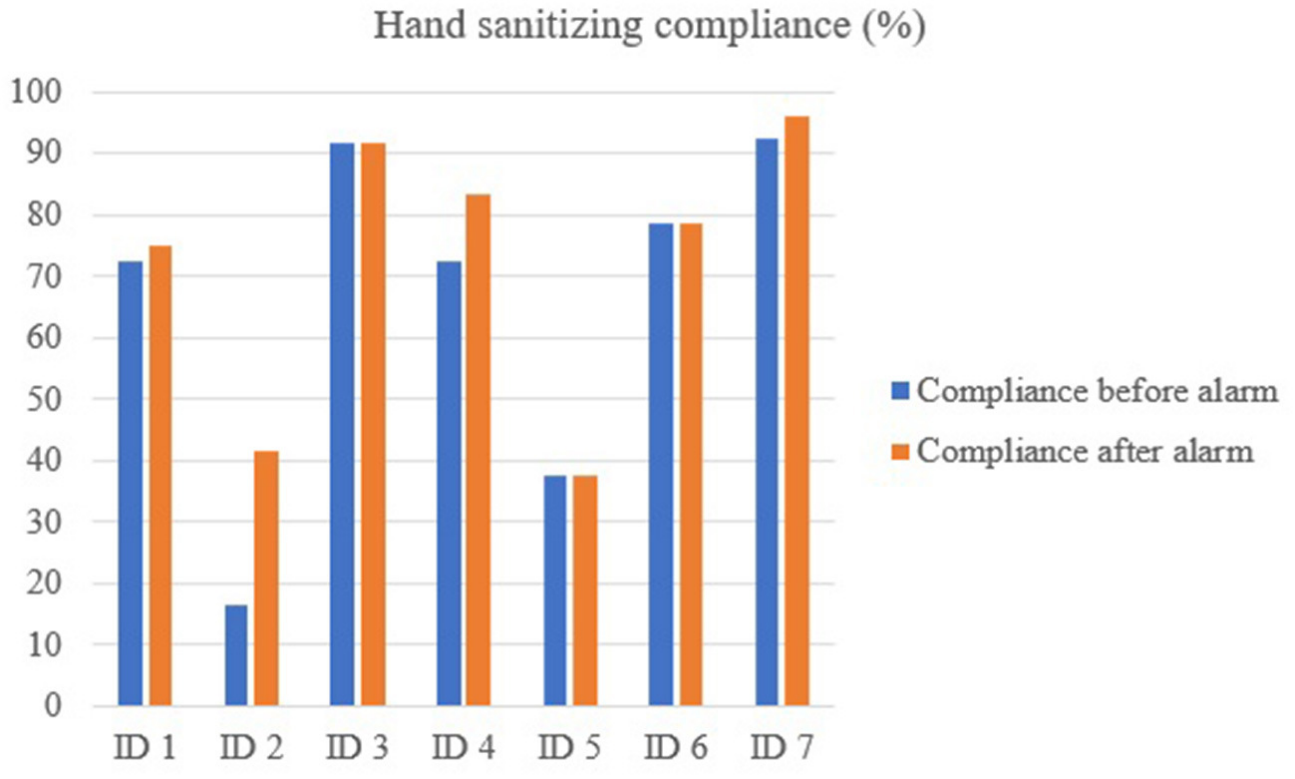
Hand sanitizing compliance rate at the individual level using the *MediHandTrace* system; before and after hearing the alarm.

Compliance rates at the farm level are presented in Figure 2. Compliance rates at the individual level are presented in Figure 3 for hand sanitizing. Table 1 presents the results of the questionnaire administered to participants.

**Table 1:** Answers provided by volunteered participants from Farm 1 and 2 of the *MediHandTrace* trial (1 being strong disagreement and 5 being strong agreement with the statement).

**Statement**	**Participant from Farm 1**			**Participant from Farm 2**		**Median answer**
	**1**	**2**	**3**	**4**	**5**	
I enjoyed participating in the experience.	4	5	4	4	4	4
I was inconvenienced by wearing chipped shoes.	1	1	2	3	2	2
The experience has allowed me to improve the application of biosecurity rules on a daily basis.	4	4	5	4	4	4
I appreciated that an alert was issued during a biosecurity breach.	5	5	5	2	4	5
I found the hand washing and shoe changing routine easy to execute.	4	4	5	4	4	4
In the context of the experiment, I found the wearing of chipped shoes intrusive to my personal life.	1	1	1	3	3	1
The location of the antennas and the alcohol-based sanitizer dispenser made it easy for me to follow the rules.	4	5	5	2	2	4
The poster describing the procedure to follow, supported by a descriptive diagram, allowed me to easily apply the rules to be followed.	4	4	4	4	4	4
I found it relevant that, when a biosecurity rule is broken, an alert is issued.	5	5	5	4	5	5
I found it relevant to evaluate the frequency of hand washing when entering and leaving the barn.	5	5	5	4	4	5
I found it relevant to evaluate if farm boots were changed during a barn visit.	5	5	5	4	5	5
The experience makes it possible to draw a real portrait of the application of biosecurity rules on the farm.	4	5	5	4	4	4
On a larger scale, the alert and monitoring system will improve compliance with biosecurity measures.	5	5	5	4	4	5

### Pilot project using the Maximus prototype

The first trial using the *MediHandTrace* system allowed for the identification of strengths and weaknesses for that system under barn conditions. With this information, the research team contacted a local private company, *Maximus* (Saint-Bruno-de-Montarville, Quebec, Canada; https://www.maximus-solution.com/en-ca), known for developing farm environmental control technologies, so that they could design a new prototype. System requirements and scenarios were given to the company engineers and a prototype was ready to be tested in 2021. The newly designed barn-adapted RFID-based system was designed to monitor farm boot and hand sanitizing compliance at the individual level when entering and leaving a barn.

**Figure 4:**
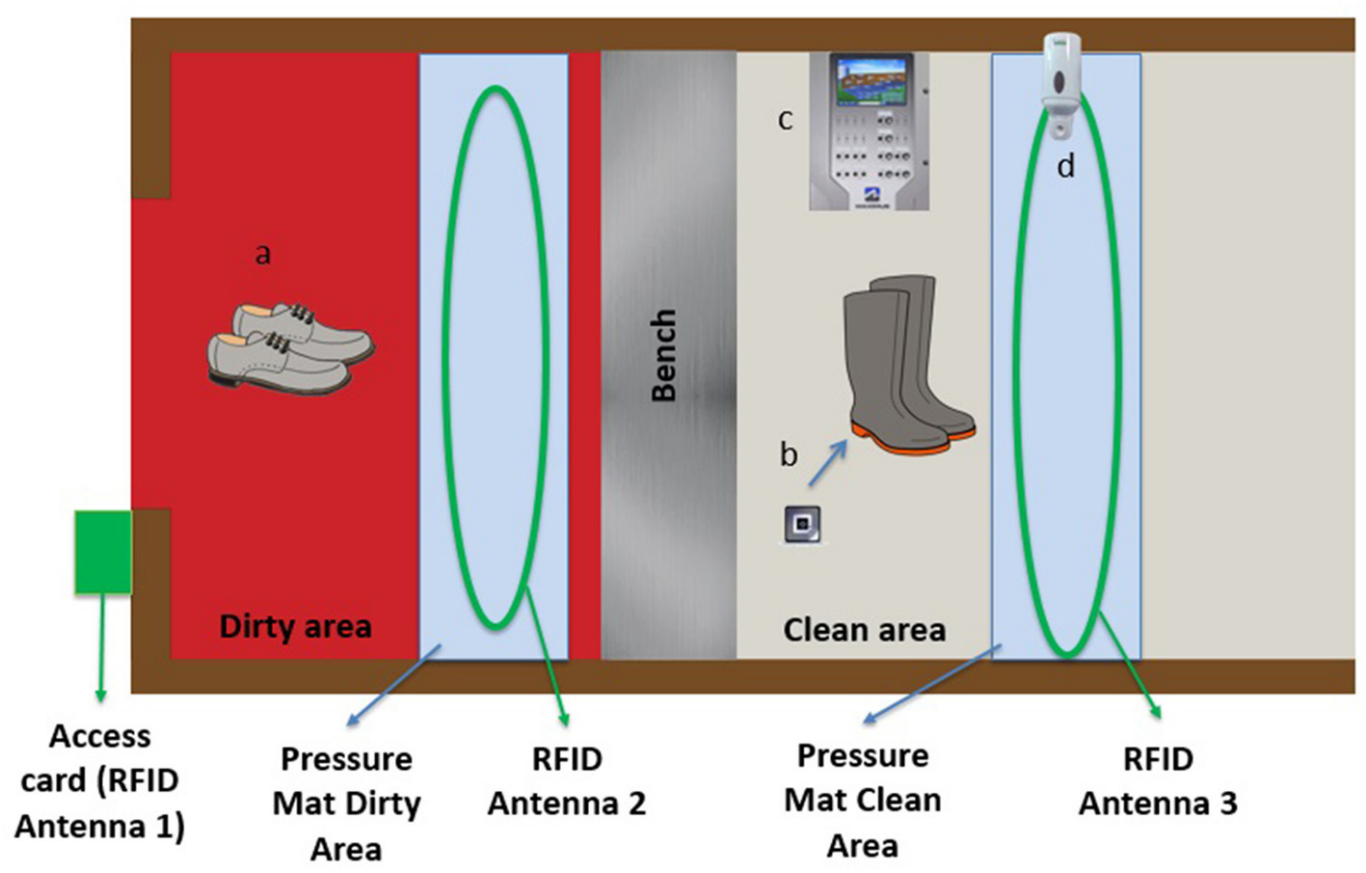
*Maximus* barn-adapted Radio-frequency-identification-based (RFID) real-time continuous automated monitoring system. **(a)** personnel work shoes without microchips; **(b)** RFID chips placed under the soles of farm boots; **(c)** Maximus control device; **(d)** hand sanitizer device.

The general objective of this second pilot project was to confirm the validity of the prototype, a RFID system equipped with three antennas and two pressure mats (Figure 4). The frequency of hand sanitizing and changing boots considering the dirty and clean areas recorded by the prototype were compared and correlated with the frequency recorded by a camera installed in the barn entrance to define the specificity of the system. The trial used the same sampling approach and inclusion criteria as the previous trial. Another ethic certificate was granted considering the use of video surveillance (Université de Montréal ethic certificate #2021-1221).

Farm boots were equipped with RFID chips (placed under the soles) for the duration of the pilot project. Three antennas in the barn entrance were connected to the prototype: the first in the frame of the exterior door identifying the person entering the barn (access card already used by employees), the second in the dirty area, allowing for the detection of chipped farm boots (indicating biosecurity non-compliance), and the third in the clean area, connected to the hand sanitizer, detecting hand sanitization when entering and leaving the clean area as well as the RFID chips in farm boots (indicating compliance with changing boots). Two pressure mats were also installed in the barn entrance (one in the dirty area and one in the clean area) to facilitate the detection of visitors, assess the direction of people (i.e., entering vs. leaving) and detect lack of compliance if farm boots were recorded in the clean area (Figure 4).

The on-farm biosecurity automated monitoring system was installed on a turkey farm in Quebec, Canada for 1one month. The barn had a three zone entrance (dirty, intermediate and clean). The system was installed on December 10 2021, and data were collected from that date until January 10 2022. For the first 2 weeks, no feedback on biosecurity breaches was made by the prototype to avoid annoying employees with alarms in case adjustments to the prototype were needed. However, we did record compliant/non-compliant events during these 2 weeks. For the last 2 weeks, the alarm system was activated and were providing real-time feedback when biosecurity breaches occurred. The prototype had a data management program allowing data collection *via* a secure Cloud system. The camera had Wi-Fi access for real-time video viewing and had a storage capacity for archiving activities in the anteroom.

**Figure 5:**
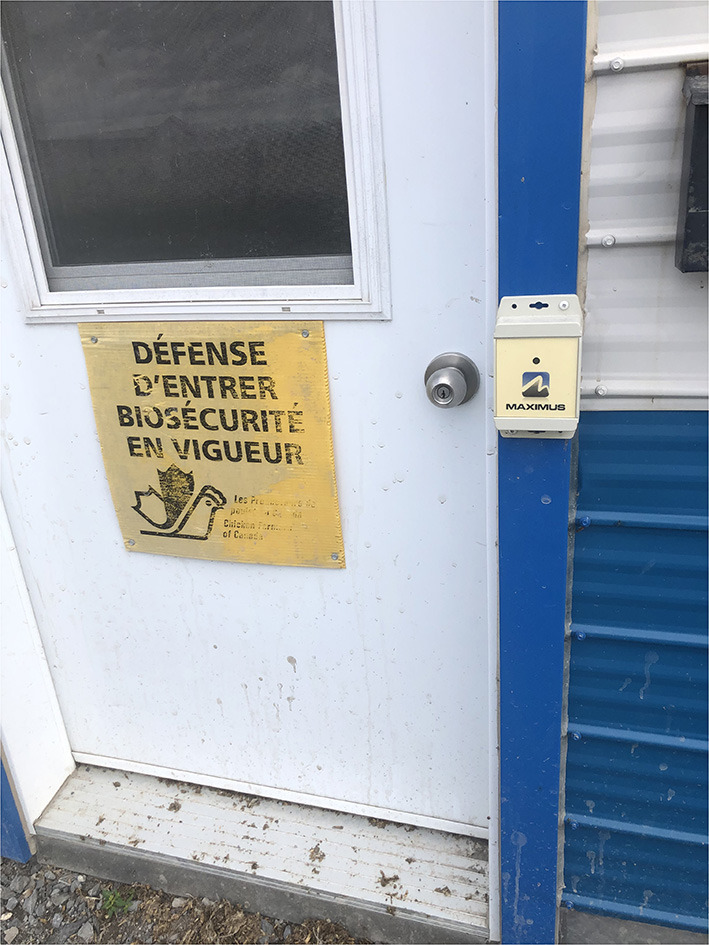
First antenna in the frame of the exterior door identifying the person entering the barn using the Maximus prototype.

**Figure 6:**
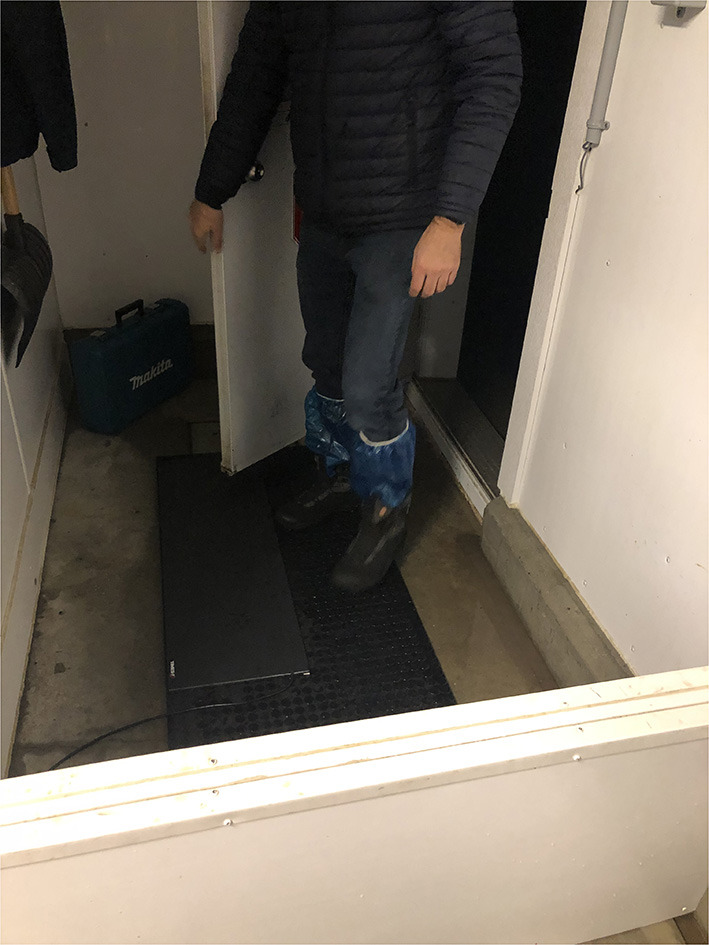
Dirty area of the turkey barn (pressure mat and antenna located on the floor)—Maximus prototype.

**Figure 7:**
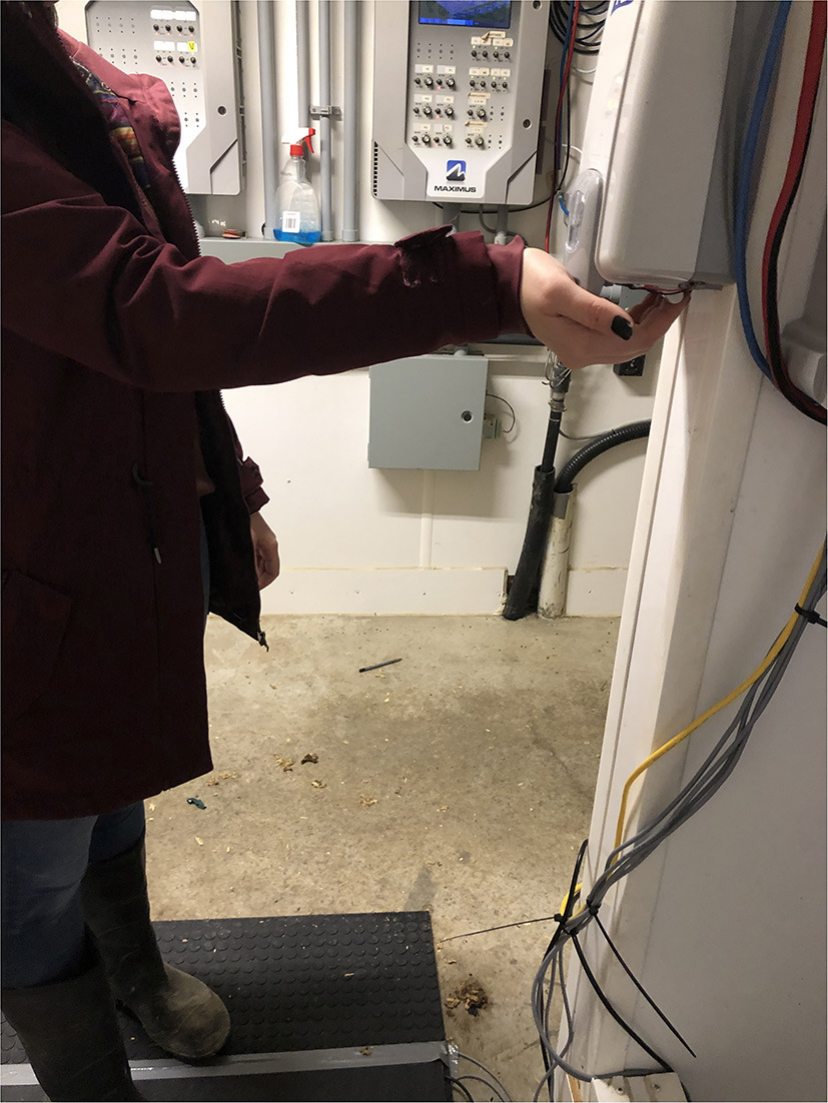
Clean area of the turkey barn (pressure mat and antenna located on the floor connected to the hand sanitizer device)—Maximus prototype.

Seven employees and four visitors took part in the study. Overall, 105 visits were recorded, 73 during week days and 32 during weekends. Visits in the morning accounted for 57% of all visits (60 in the morning, 45 in the afternoon). Employees did on average 15 visits each during the study period according to their access cards read by antenna 1 (Figures 4, 5). The camera detected 105 entrances, while the Maximus system detected 81 (capacity of detection of 77%). The main challenge was to record multiple people entering at the same time (Figure 6). The camera recorded 104 exits (one person exited by another door), while the Maximus system detected 163 exits (overestimation of 57%), mainly due to the barn entrance design. Indeed, the three-zone entrance was L-shaped and the clean area was located in a corridor where traffic between two rooms was common (Figure 7). As the pressure mat and the antenna were located in this corridor, the system recorded these passages as exits leading to an overestimation. Indeed, although the coverage by the antenna was limited to the anteroom, it was extended enough to create problems when the design of the anteroom makes it possible to pass nearby while going between the two rooms. This can be prevented by limiting (e.g., dedicated space, delimitation using a physical barrier, etc.) the traffic nearby the antenna and pressure mat or by programming the system to conclude on an exit once a person is detected on the pressure mat in the dirty area (Figure 6).

**Figure 8:**
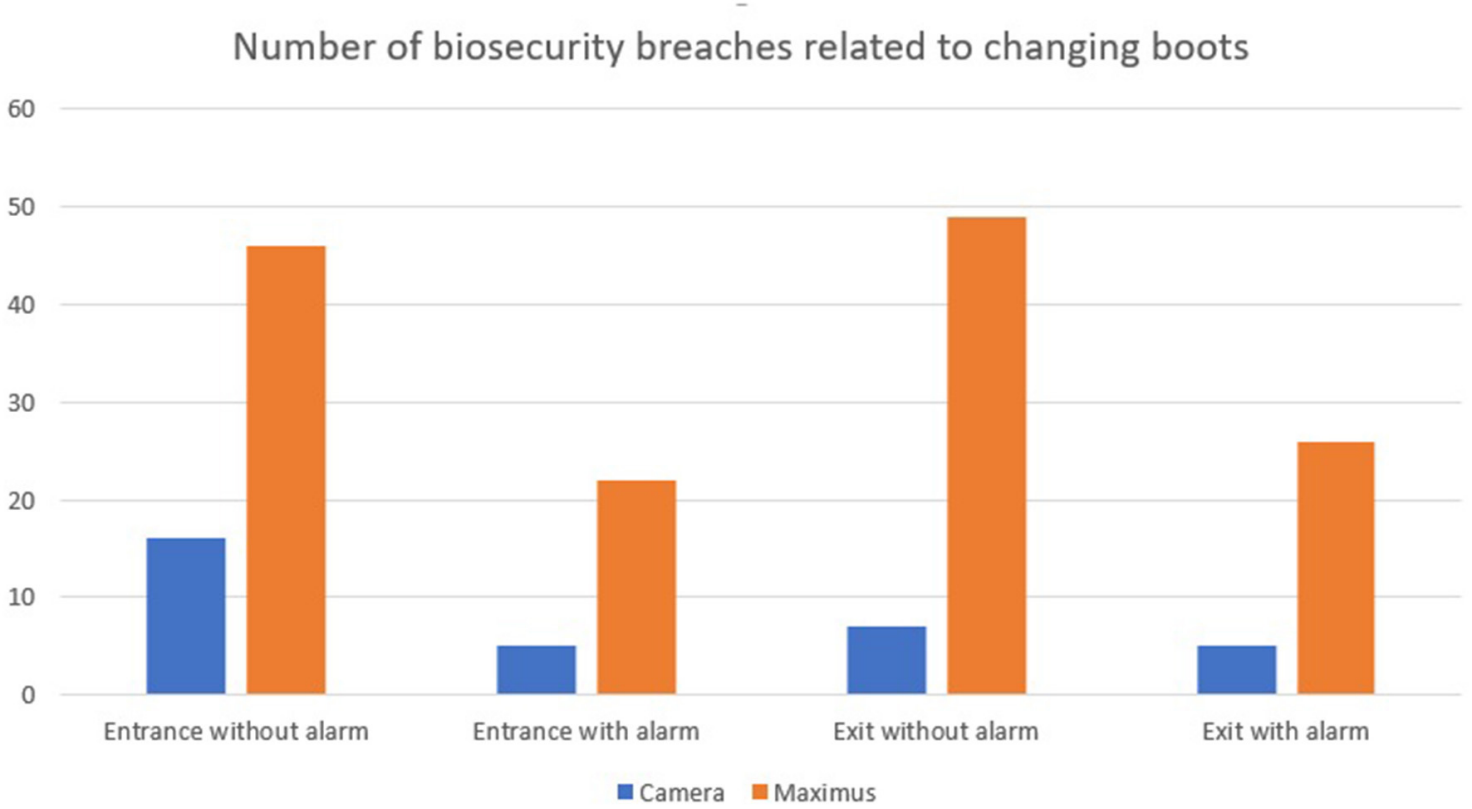
Number of biosecurity breaches related to changing boots recorded by the camera vs. the Maximus system (with and without the alarms).

Detections of non-compliance with biosecurity measures were calculated as per the camera installed in the barn entrance and the RFID system. When entering the clean area, the camera recorded 21 (20 %) non-compliant farm boot events, i.e., the person did not don farm boots, 16 in the first 2 weeks (without alarm); 5 in the last 2 weeks (with alarms). The prototype recorded 68 (64.8%) non-compliant events, i.e., a person was detected by the pressure mat in the clean area, but the RFID chip was not detected by antenna #3 [46 in the first 2 weeks (without alarm); 22 in the last two weeks (with alarms)]. The discrepancy was attributed to antenna #3 not being sensitive enough to detect RFID chips inserted in farm boots. This problem can be easily adjusted. In addition, only one farm boot for a given pair was chipped. Chipping both boots should increase detection. Figure 8 presents the number of biosecurity breaches related to changing boots recorded by the camera vs. the Maximus system when entering and leaving the barn for the first 2 weeks (without the alarm) and the last 2 weeks (with the alarms).

When entering the clean area, the camera recorded 53 (50%) non-compliant hand sanitizing events, 35 in the first 2 weeks (without alarm); 18 in the last 2 weeks (with alarms). The prototype recorded 89 (85%) non-compliant events, 60 in the first 2 weeks (without alarm); 29 in the last 2 weeks (with alarms). When leaving the barn, the camera recorded 60 (57.7%) non-compliant hand sanitizing events, 41 in the first 2 weeks (without alarm); 19 in the last 2 weeks (with alarms). The prototype recorded 95 (91.3%) non-compliant events, 59 in the first 2 weeks (without alarm); 36 in the last 2 weeks (with alarms). The discrepancy was attributed to the fact that the prototype was programmed for a two-area barn entrance design and it was expected that hands would be sanitized in the clean area when entering and leaving a barn. The selected barn had a three-zone entrance for which hands are expected to be sanitized in the intermediate area (before moving to the clean area when entering the barn and before moving to the dirty area when leaving). Therefore, the prototype overestimated hand sanitizing non-compliance. This can be fixed by allowing hands to be sanitized before detection of a person on the pressure mat located in the clean area when entering the barn and after detection on the pressure mat when leaving the barn.

## Discussion

Results from the *MediHandTrace* system confirmed the ability of the system to monitor boot and hand sanitizing compliance. Having a continuous monitoring system seems to improve biosecurity compliance with almost twice the compliance percentage compared to previous studies using hidden cameras (21). However, part of this improved compliance level could be due to the fact that participants were made aware of the objectives of the project and it was only a short term assessment. In other words, the percent compliance would likely be less 6 months after the initiation of the project. This was observed by Racicot et al. (23). Still, participants reported that the RFID system was helping increasing daily compliance, and recommended using it on a larger scale to improve overall compliance. They reported being comfortable with shoes being chipped and did not find the project intrusive. However, the sample size of the survey was limited. The survey was mainly designed to have an idea of the system acceptability before investing time, money and effort in manufacturing a farm-adapted system. As participants were generally in favor, the Maximus prototype was worked on.

Although not used during the pilot phase of this project, the *MediHand Trace* system was also able to provide feedback to employees at a desired frequency by SMS. Comparing performance with peers was also another interesting functionality, not used during this project. These functionalities would be contributing to motivating employees by providing personal performance and peer comparison data and should be used to reward good compliance. It is recognized that peer comparison increases compliance (35, 36). These functionalities could also be used to evaluate the effectiveness of biosecurity training programs. However, providing real-time feedback seems to have a variable impact on the participants. Some employees were highly influenced by the alarm and the feedback mechanism helped correcting their behavior, while others were not influenced at all. Psychological characteristics, such as personality traits (22), are closely linked to biosecurity compliance. It may also be the case for the receptivity to real-time audio feedbacks.

This system designed for hospitals had limitations for on-farm usage. Because it only had one antenna installed in the clean area, it was not able to detect farm boots in the dirty area, and so could not record this type of non-compliance (e.g., removing farm boots in the dirty area or leaving the barn with farm boots). In addition, the system was not able to detect if someone was going back and forth in the clean area or leaving the barn, resulting in potentially hand sanitizing non-compliance overestimation. Furthermore, we had to assume that participants would always use the personal footwear that they agreed to be set with a chip when coming to work. In the situation where participants would have worn unchipped personal footwear and would not have followed the biosecurity rules, the RFID system would not have detected this visit and would not have recorded any information on compliance, which is a significant limitation. Pressure mats are needed to detect when people enter and leave the barn, instead of relying on chipped personal footwear to be detected. Finally, visitors were difficult to monitor as most would likely only wear disposable plastic boots, which are not chipped, thus not detected by the antenna. Consequently, the system had limited capacity in monitoring visitors' compliance.

The trial with the *MediHand Trace* system was useful to design the Maximus prototype with the objective to have a more in-depth compliance monitoring by adding pressure mats and antenna in the dirty area. However, as we fixed the limitation associated with having no antenna in the dirty area, we added a challenge, since the extra antenna cannot simply be located in the dirty area without considering the design of each barn anteroom. Farm 1 was an egg layer farm with a barn entrance well-designed (corridor-type). Farm 2 was a broiler farm with a barn entrance with significant space limitation to display the antenna and hand sanitizer. The corridor-type barn entrance was more suitable for the RFID system and was not impacting the daily biosecurity routine, while the second farm was not as intuitive.

Even if the initial Maximus prototype did not perform as expected, the trial allowed to better understand the impact of the barn entrance design in monitoring compliance and make adjustments accordingly. Indeed, as biosecurity compliance was monitored using cameras to compare with biosecurity breaches recorded by the RFID prototype, it was possible to identify the system limitations after the first tested farm and apply changes for upcoming farms. As seen with the *MediHand Trace* trial, it was possible to notice that biosecurity breaches were lower when alarms were activated on the Maximus prototype. Participants reacted each time the alarm sounded, often wondering what they may have done wrong. As biosecurity protocols in barn entrance are similar on poultry and swine premises, it is expected that the next trials will be performed on swine farms.

## Conclusion

Monitoring biosecurity is needed to maintain compliance. Relying on video surveillance is too burdensome and expensive to be largely implemented. However, relying on new technologies will improve biosecurity compliance and reduce the likelihood of introduction and spread of infectious diseases. Having evidence-based results will help convincing producers of the importance of these measures and of providing feedback for maintaining biosecurity compliance. These pilot studies have shown that real-time feedback helps improve compliance. However, the efficacy of the systems was very dependent on the physical set-up of the anteroom. An implementation of the technology by producers will allow us to collect long-term data without the potential bias associated with assessments following instructions.

## Data availability statement

The raw data supporting the conclusions of this article will be made available by the authors, without undue reservation.

## Ethics statement

The studies involving human participants were reviewed and approved by Université de Montréal Ethics Committee (certificate #2018-688, CERES-18-097-D). The patients/participants provided their written informed consent to participate in this study.

## Author contributions

MR had the original idea and produced the initial design of the pilot studies, including adapting the *MediHand Trace* system to a barn environment, recruiting the company Maximus for the design of their prototype, helped creating the prototype and supervised the technician and the student working in the field, and also produced the first draft of the paper. J-PV provided the funding for the purchase of the *MediHand Trace* system, recruited and covered the salary of the veterinary student and assisted with her supervision, and also covered the expenses associated with the technician, contributed to the design of the Maximus prototype and edited the article, adding to its different sections. DT and A-MC conducted the field work, collected the data, contributed to adjustments for the two systems, identified limiting factors, and proposed solutions. All authors contributed to the article and approved the submitted version.

## Conflict of interest

The authors declare that the research was conducted in the absence of any commercial or financial relationships that could be construed as a potential conflict of interest.

## Publisher's note

All claims expressed in this article are solely those of the authors and do not necessarily represent those of their affiliated organizations, or those of the publisher, the editors and the reviewers. Any product that may be evaluated in this article, or claim that may be made by its manufacturer, is not guaranteed or endorsed by the publisher.
